# Data on known anti-virals in combating CoVid-19

**DOI:** 10.6026/97320630016878

**Published:** 2020-11-30

**Authors:** L Akshayaa, Thangavelu Lakshmi, Ezhilarasan Devaraj, Anitha Roy, S Raghunandhakumar, P Sivaperumal, Sheba David, Kamal Dua, Dinesh Kumar Chellappan

**Affiliations:** 1 Department of Pharmacology, Saveetha Dental College, Saveetha Institute of Medical and Technical Sciences, Saveetha University, Chennai-600077, TamilNadu; 2 PAPRSB Institute of Health Sciences, Universiti Brunei Darussalam, Brunei; 3 Discipline of Pharmacy, Graduate School of Health, University of Technology Sydney, Ultimo, NSW, 2007, Australia; 4 School of Biomedical Sciences and Pharmacy, University of Newcastle, Newcastle NSW 2308,Australia; 5 Department of Life sciences, School of Pharmacy, International Medical University ,Bukit Jalil, 57000, Kualalumpur, Malaysia

**Keywords:** COVID -19, antiviral, ivermectin, remdesir, SARS-Cov2

## Abstract

Design and development of effective anti-virals in combating CoVid-19 is a great challenge worldwide. Known drugs such as chloroquine, lopinavir, favipiravir and remdesivir are used in the management of CoVid - 19. It is known that Ivermectin and remdesivir both are effective against filoviruses, paramyxo viruses. Available data also shows that ivermectin and remedesivir repress the replication of SARS-CoV-2. Thus, we document the potential use of ivermectin and remdesivir in the management of CoVid -19.

## Description:

The clinical highlights of COVID-19 infection are sore throat, shortness of breath, fever, cold, Severe Acute Respiratory Infection (SARS) [1,2]. Antivirals including interferon alpha, lopinavir, ritonavir, hydroxychloroquine, ribavirin and arbidol have been introduced by the National Health Commission (NHC), which goes as the rule for the counteraction, analysis, and treatment of Novel Coronavirus-actuated Pneumonia [3]. The potential impact of antiviral medication in patients with COVID-19 is assessed utilizing randomised, placebo-controlled investigations [4,5]. A study indicated that remdesivir found to inhibit replication of a wide spectrum of coronaviruses and MERS-CoV invitro [6]. A few clinical studies have reported that ivermectin, remedesivir, found to repress the replication of SARS-CoV-2 in vitro [7]. Ivermectin has been recognized as a high wellbeing profile for human use with expanded medication dosing dependent on its pharmacokinetic activity [8]. Both invivo and invitro analysis on remdesivir reported that a single portion of ivermectin shows a strong inhibitory activity against DNA in pseudorabies viral infection [9,,10]. The activity of ivermectin found to restrain the DNA polymerase, which has been deductively assessed in vitro study [11]. An ongoing investigation on clinical treatment have observed that an oral portion of ivermectin brought about a critical decrease in the serum level of viral NS 1 protein in dengue infections [12]. A previous study done, to test the viable blend of both ivermectin and ridamsavir, has been reported that these medications will diminish the viral RNA by 99.8% in 24 hrs in vitro premise [13]. Remdesivir is a potent immunomodulator against a wide range of infections caused by filoviruses, coronaviruses [14]. An accomplice study done on hospitalized patients with serious pneumonia, shows clinical improvement when administered with antiviral remedesivir [15]. Previous in vitro study reported by Mulangu S et al, shows positive adequacy against bat nCov and human SARS related respiratory infections [16]. A comparative investigation done by Warren et al. showed that the intravenous administration of 10 mg/kg portion of remdesivir in Non human primate model (NHP) brought about 100% protection against Ebola infection disease [17]. It has been demonstrated that remedesivir acts as the potential medication for the treatment of Covid-19 infection [18,19]. Remdesivir as a combination with other antiviral medications found to hinder human endemic and zoonotic delta infections with a profoundly dissimilar RNA polymerase enzyme [20]. A clinical investigation announced by De weit et al, reported that prophylactic treatment of ramdesivir found to diminish the irritation of lungs in COVID- 19 influenced patients [21]. A comparative clinical investigation revealed that there is an equipotent cytotoxic impact shown by the microorganisms and human beings [22]. It is also revealed that utilization of both ivermectin and remdesivir effective for the treatment of lower respiratory tract infections [23]. Thus, available data also shows that ivermectin and remedesivir repress the replication of SARS-CoV-2. Thus, we document the potential use of ivermectin and remdesivir in the management of CoVid -19.

## References

[1] Bai Y (2020). JAMA.

[2] Mehta M (2019). Chemico-Biological Interactions.

[3] Wang D (2020). The Journal of infection.

[4] Yavuz SS, Unal S (2020). Turk J Med Sci.

[5] Sharma P (2019). Chemico-Biological Interactions.

[6] Caly L (2020). Antiviral research.

[7] Muñoz J (2018). PLoS neglected tropical diseases.

[8] Lv C (2018). Antiviral research.

[9] Lakshmi T (2015). Pharmacognosy Reviews.

[10] Furuta Y (2017). Physical and Biological Sciences.

[11] Guo YR (2020). Military Medical Research.

[12] Buonfrate D (2019). The Lancet infectious diseases.

[13] Agostini ML (2018). mBio.

[14] Grein J (2020). The New England journal of medicine.

[15] Mulangu S (2019). The New England journal of medicine.

[16] Warren TK (2016). Nature.

[17] Wang M (2020). Cell research.

[18] Menon S (2018). Colloids Surf B Biointerfaces.

[19] Brown AJ (2019). Antiviral research.

[20] de Wit E (2020). Proceedings of the National Academy of Sciences of the United States of America.

[21] Zhou Y (2020). Cell Discovery.

[22] Uyeki TM (2014). The New England journal of medicine.

[23] Holshue ML (2020). The New England journal of medicine.

